# Prevalence of variations in melanoma susceptibility genes among Slovenian melanoma families

**DOI:** 10.1186/1471-2350-9-86

**Published:** 2008-09-19

**Authors:** Barbara Peric, Petra Cerkovnik, Srdjan Novakovic, Janez Zgajnar, Nikola Besic, Marko Hocevar

**Affiliations:** 1 Department of Surgical Oncology, Institute of Oncology Ljubljana, Zaloška 2, SI-1000, Ljubljana, Slovenia

## Abstract

**Background:**

Two high-risk genes have been implicated in the development of CM (cutaneous melanoma). Germline mutations of the CDKN2A gene are found in < 25% of melanoma-prone families and there are only seven families with mutation of the *CDK4 *gene reported to date. Beside those high penetrance genes, certain allelic variants of the *MC1R *gene modify the risk of developing the disease.

The aims of our study were: to determine the prevalence of germline *CDKN2A *mutations and variants in members of families with familial CM and in patients with multiple primary CM; to search for possible *CDK4 *mutations, and to determine the frequency of variations in the *MC1R *gene.

**Methods:**

From January 2001 until January 2007, 64 individuals were included in the study. The group included 28 patients and 7 healthy relatives belonging to 25 families, 26 patients with multiple primary tumors and 3 children with CM. Additionally 54 healthy individuals were included as a control group. Mutations and variants of the melanoma susceptibility genes were identified by direct sequencing.

**Results:**

Seven families with CDKN2A mutations were discovered (7/25 or 28.0%). The L94Q mutation found in one family had not been previously reported in other populations. The D84N variant, with possible biological impact, was discovered in the case of patient without family history but with multiple primary CM. Only one mutation carrier was found in the control group. Further analysis revealed that c.540C>T heterozygous carriers were more common in the group of CM patients and their healthy relatives (11/64 vs. 2/54). One p14ARF variant was discovered in the control group and no mutations of the *CDK4 *gene were found.

Most frequently found variants of the *MC1R *gene were T314T, V60L, V92M, R151C, R160W and R163Q with frequencies slightly higher in the group of patients and their relatives than in the group of controls, but the difference was statistically insignificant.

**Conclusion:**

The present study has shown high prevalence of p16INK4A mutations in Slovenian population of familial melanoma patients (37%) and an absence of p14ARF or *CDK4 *mutations.

## Background

Cutaneous melanoma (CM) is the most common cause of death from skin cancer [1]. According to epidemiology studies, the risk of CM is influenced by genetic and environmental factors. Exposure to UV radiation is the main environmental factor, while the genetic basis of CM is complex and appears to involve multiple genes. It is now widely recognized that autosomal dominant inheritance of certain genes is associated with predisposition to CM. Two high penetrance melanoma susceptibility genes, *CDKN2A *and *CDK4*, and a low penetrance gene, *MC1R*, have been identified [2].

*CDKN2A *is a tumor suppressor gene encoding two distinct proteins from alternatively spliced transcripts [3]. One of those proteins is p16INK4A, which inhibits the CDK4/6 mediated phosphorilation and inactivation of Rb (retinoblastoma) protein. The alternative reading-frame product – p14ARF inhibits the MDM2-mediated ubiquitylation and subsequent degradation of p53 protein [4]. Mutations affecting only the p14ARF protein product are rare.

Germline *CDKN2A *mutations have been detected in 10–25% of melanoma-prone families from North America, Europe and Australia [5]. Studies in the past have also indicated an increased risk of pancreatic cancer (PC) in families with *CDKN2A *mutations [6].

In contrast to numerous cases of *CDKN2A *mutations in families with CM, to our knowledge only seven families with *CDK4 *germ-line mutations have been identified worldwide [7]. The *CDK4 *gene is an oncogene encoding cyclin dependent kinase 4. All reported *CDK4 *mutations are located in exon 2, which codes for the p16INK4A binding site [3].

The gene encoding melanocortin 1 receptor protein (*MC1R*) does not play a part in cell cycle regulation. It is expressed on epidermal melanocytes, functioning as a binding receptor for alpha melanocyte-stimulating hormone (MSH). MC1R-MSH complex induces cyclic-AMP tyrosinase activity and melanin production [2]. The *MC1R *gene determines the type of melanin produced in the skin, but its role in melanoma carcinogenesis reaches beyond its pigmentation effects. *MC1R *variants R151C, R160W and D294H are associated with a skin phenotype prone to the development of CM (red hair, fair skin, freckles and an inability to tan). These variants are known as RHC (red hair color) variants and are associated with an increased risk of developing CM independently of skin phenotype. It is believed that they also act as modifiers of CM risk in carriers of *CDKN2A *mutations [8-10].

Slovenia has a geographical latitude of 46°02' and two million inhabitants. The reported incidence rate of CM was 14.5 per 100 000 for males and 14.9 for females in the time period 2000 to 2004. In the year 2006, the expected incidence rate was 16 per 100 000 [11].

The major aims of our study were: i) to determine the prevalence of germline *CDKN2A *mutations and variations in members of families with familial CM and in patients with multiple primary CM; ii) to search for individuals/families with possible *CDK4 *mutations; iii) to determine the frequency of *MC1R *gene variants and to correlate them with development of CM in the population of patients from melanoma families and their healthy relatives. In an extended genotyping, we also looked for the same genetic alterations among healthy individuals belonging to the control group.

## Methods

### Patients

From January 2001 to January 2007, 64 individuals were included in this study of melanoma susceptibility genes, after being referred to the Institute of Oncology Ljubljana for further treatment or genetic counseling. The group comprised 28 patients and 7 of their healthy relatives belonging to 25 melanoma families, and 26 patients with multiple primary CM. Melanoma families were defined as: (a) having at least two affected family members, or (b) a case of PC in a first-degree relative. In two cases family history was based on a second-degree relative with CM. All included unaffected members of the melanoma families were patient's first degree relatives. Additionally, 3 children diagnosed with CM at eight, nine and twelve years were included. They were not members of melanoma families. In all cases the diagnosis of CM was confirmed by pathology report and family histories were obtained from personal interviews. Additional cancers that were reported in this study were colon cancer (three families), lung cancer (two families) and breast, prostatic and non-melanoma skin cancer (each in one family). All adult patients, and the parents of the three children, were informed about the genetic research protocol and signed a consent form agreeing to donate blood samples. The study was reviewed and approved by the Slovenian National Medical Ethics Committee. Table 1 contains demographic details about the study population.

**Table 1:** Demographic details

**Study population (n = 64)**						
	***Family history***	***Unaffected relatives***	***Multiple primary CM***	***Child***	***Gender***	
					***female***	***male***
No	28	7	26	3	41	23
Age at the time of diagnosis (years) of 57 patients*						
Pts No (%)		< 15	< 45		> 45	
		3 (5.3%)	25 (43.8%)		29 (50.8%)	
***Families (n = 25)***						
		***< 3 affected members***	***≥ 3 affected members***		***CM and PC***	
No of fam.		17	4		4	

* the 7 healthy relatives are not included

### Healthy controls

Randomly chosen individuals were invited to donate blood samples at the Institute of Oncology Ljubljana. Individuals previously diagnosed with cancer were excluded. All participants agreed that their blood sample can be used to create a DNA bank, which could be repeatedly used in various genetic researches, but they will not be informed about the research results. 54 DNA samples of volunteers with comparable sex and age were chosen from that bank in order to match the individuals in the group of patients and their relatives. The group included 28 females (aged 24 – 70 years) and 26 males (aged 24 – 63). At the time of study, 48.1% of individuals were under 45 years and 51.8% of individuals were older than 45 years.

### Mutation screening of melanoma susceptibility genes

For mutation analysis, genomic DNA was isolated from 10 ml of peripheral blood using QIAamp^® ^DNA Blood Maxi kit (Qiagen, Hilden, D) according to the manufacturer's protocol.

Exons 1α, 1β, 2 and 3, including part of promoter region and 3'-UTR region of the *CDKN2A *gene, exon 2 of the *CDK4 *gene and the coding sequence of the *MC1R *gene were amplified by polymerase chain reaction (PCR) as described previously [12-14].

Direct sequencing, using an automated fluorescence-based cycle sequencer (ABI Prism^® ^310 Genetic Analyzer, Applied Biosystems, Warrington, UK), identified mutations/variants. Chromatograms were read with the aid of ABI Prism 310 software (Applied Biosystems) and utilizing the Gene Runner software tool, version 3.05. Numbering of variants relates to the following reference sequences, *CDKN2A *gene: GeneBank accession no. AF527803 for coding region and 3'-UTR region, and X94154 for promoter region, *CDK4 *gene: GeneBank accession no. AF507942 and *MC1R *gene: GeneBank accession no. AF263461.

### Statistics

Pearson Chi-Square test was used to compare the number of different variant carriers between the patients and the control group.

Student's t test was used for mean age comparison of patients who were p16INK4A mutation-positive and those who were mutation-negative.

Allele frequencies of *MC1R *variants were defined as the percentage of times that a given allele is present at a locus within each tested group and compared with Fisher's exact test. All statistical analyses were performed with the Statistical Package for Social Sciences (SPSS) v. 15.0. P-values less than 0.05 were considered statistically significant.

## Results

Sequencing of the 1α, 2 and 3 *CDKN2A *exons revealed 11 different allelic variants presented in Table 2. There were 7 mutations (named p16INK4A mutations), one variant of the coding region, one variant of 5'UTR and two variants of 3'UTR. In all but one case, the mutations were discovered in the group of patients with positive family history or their relatives (14/35). One mutation (D153E) was found only in the group of healthy control individuals. In the same group, we found one case of a A148T variant, which was also found in one case of familial melanoma and in two cases of multiple primary CM. Altogether three patients with multiple primary CM had two different allelic variants of the coding region (two cases of A148T and one of D84N variant, 3/26). Analysis of the samples taken from the three children revealed no changes in the coding region of *CDKN2A *(Table 2), but it showed that they were all heterozygous for the same 5'UTR and 3'UTR variants. Three out of four families with ≥ 3 cases of CM were mutation-positive (3/4).

**Table 2:** CDKN2A variants among individuals with family history, patients with multiple primary CM, and healthy controls

			**POSITIVE CASES**					
			
**Location**	**CDKN2A ** **nucletide** **change**	**Aminoacid ** **change**	***Pts with *** ***family history*** ***(28)***	***Healthy*** ***relatives*** ***(7)***	***Families *** ***(25)***	***Pts with multiple*** ***primary only*** ***(26)***	***Children *** ***(3)***	***Healthy*** ***controls*** ***(54)***
Exon 1α	68G > A	G23D	2	1	2	0	0	0
Exon 1α	71G > C	R24P	1	1	1	0	0	0
Exon 1β	69C > T	F23F	0	0	0	0	0	1
Exon 2	250G > A	D84N	0	0	0	1	0	0
Exon 2	281T > A	L94Q	3	2	1	0	0	0
Exon 2	301G > T	G101W	2	0	2	0	0	0
Exon 2	442G > A	A148T	1	0	1	2	0	1
Exon 2	459C > A	D153E	0	0	0	0	0	1
Intron 1	IVS1-1G > A		1	0	1	0	0	0

In the group of patients and their relatives, 38 individuals had the -191Met A > G variant of 5'UTR (38/64) compared to 31 cases in the control group (31/54). In both groups, most of the cases were heterozygous. Sequencing of 3'UTR gave similar results considering the IVS3+29C > G (c.500C > G) variant (22/64 vs. 13/54). Comparing both groups for another allelic variant showed that IVS3+69C > T (c.540C > T) heterozygous carriers were more common in the group of patients with CM and their healthy relatives (11/64 vs. 2/54). Results are presented in Table 3. There were no differences in the distribution of variants considering family history or multiple primary CM.

**Table 3:** Variants in 5'- and 3'-UTRs

	**3'UTR and 5'UTR VARIANTS**		
	**Positive cases**		
***Location***	***CDKN2A *** ***nucletide*** ***change***	***Pts and healthy *** ***relatives*** ***(n = 35)***	***Healthy *** ***controls*** ***(n = 54)***

3'-UTR	500C > G	22	13
3'-UTR	540C > T	12	2
5'-UTR	-191A > G	38	31
		Pts with CM (n = 28)	Healthy controls (n = 54)
500C > G	heterozygous	17	12
	homozygous	2	1
540C > T	heterozygous	11	2
	homozygous	1	0
-191A > G	heterozygous	26	28
	homozygous	8	3

Sequencing of the exon 1β revealed no mutations in the group of patients with CM or their healthy relatives. In the control group, we found the F23F allelic variant (numbered from the second start codon).

At the time of diagnosis, mean age of patients who were p16INK4A mutation-positive was 38.40 years compared to 45.11 years for mutation-negative patients (p = 0.257).

There were no mutations found on exon 2 of the *CDK4 *gene in our population of CM patients or healthy control individuals.

Analysis of the complete *MC1R *sequence revealed 14 different variants, including "R" alleles and 1 deletion (Table 4). The most frequently found ones were T314T, V60L, V92M, R151C, R160W and R163Q. D294H was among rarely encountered variants. The deletion was found in the case of a patient with no family history, but with multiple primary CM.

**Table 4:** Distribution of MC1R variants among patients with CM and their healthy relatives and in the control group.

**No**	**MC1R variant**		**Pts and healthy relatives ** **(n = 64)**	**Allel Freq. ** **(%)**	**Control group ** **(n = 54)**	**Allel Freq. ** **(%)**	**p**
						
	***Protein ***	***Nucleotide***					
1	p. V60L	c.178G > T	11	8.6	9	8.3	1.00
2	p. V92M	c.274G > A	13	10.1	9	8.3	0.66
3	p. R142H	c.425G > A	1	0.8	0	0.0	1.00
4	p.R151C*	c.451C > T	12	9.4	6	5.5	0.32
5	p.I155T	c.464T > C	1	0.8	0	0.0	1.00
6	p.R160W*	c.478C > T	11	8.6	8	7.4	0.81
7	p.R163Q	c.488G > A	6	4.7	4	3.7	0.75
8	p.R213W	C.637C > T	1	0.8	0	0.0	1.00
9	p.A218T	c.652G > A	0	0.0	1	0.9	0.46
10	p.G239G	c.717C > T	0	0.0	1	0.9	0.46
11		c.754delC	1	0.8	0	0.0	1.00
12	p.L286V	c.856C > G	2	1.6	0	0.0	0.50
13	p.D294H*	c.880G > C	1	0.8	0	0.0	1.00
14	p.T314T	c.942A > G	14	10.9	10	9.2	0.82
15	p.S316S	c.948C > T	0	0.0	1	0.9	0.46

* "R" alleles: RHC variants were classified by strength of association with red hair into strong and weak RHC alleles. Strong alleles included p.R151C, p.R160W, p.D294H and were designated as "R".

There were 11 carriers of at least one *MC1R *variant in the group of individuals with one of the *CDKN2A *coding region allelic variants. Three healthy carriers of the p16INK4A mutation also had at least one *MC1R *variant, but in the six p16INK4A mutation carriers with CM, there were no *MC1R *variants found (Table 5).

**Table 5:** CDKN2A and MC1R allelic variants in patients with CM

CASE	CM	CDKN2A allel. variants	MC1R alleles
1	yes, multiple	p.L94Q	"R"
2	yes, multiple	p.L94Q	
3	no	p.L94Q	"r"
4	yes	p.L94Q	
5	no	p.L94Q	"R"
6	yes, multiple	p.G23D	"r"
7	no	p.G23D	"R"
8	yes, multiple	IVS1-1g>a	"R"
9	yes, multiple	p.G101W	
10	yes, multiple	p.R24P	"r"
11	yes	p.A148T	"R"
12	yes	p.G101W	
13	yes	p.A148T	
14	no	p.R24P	"r"
15	yes	p.G23D	"r"
16	yes, multiple	p.A148T	"r"
17	yes, multiple	p.D84N	

## Discussion

We analyzed melanoma susceptibility genes in the DNA samples of 64 individuals with multiple primary CM and/or family history of CM. Many of them (35/64 or 54.7%) had a family history of CM and/or PC and belonged to 25 different melanoma families. In order to avoid mistaking allelic variants commonly present in Slovenian population as variants observed in CM cases, we additionally analyzed DNA samples of 54 healthy individuals.

Since the mutation carriers belonged to 7 different CM families, the frequency of mutation-positive families was 7/25 (28.0%). Of 35 individuals with positive family history 13 (37%) were p16INK4A mutation-positive. This is similar to northern parts of neighboring Italy (25% of families with two CM cases), where frequencies vary according to each specific region [15]. Researchers from Germany, Poland and Latvia report much lower frequencies or absence of p16INK4A mutations [7,16,17]. In Utah reported frequency for melanoma families is also lower, only 8.3% – 10% in physician-referred pedigrees. One possible reason for the observed high mutation rate in our population of CM families is that their recruitment was based on physician referral. Population-based assessments usually report somewhat lower presence of *CDKN2A *mutations [1].

The percentage of p16INK4A mutation-positive families was higher in the case of families with 3 affected members, as expected [18]. In our study only 4 families had three members with CM and three of those families were p16INK4A mutation-positive (75%). In one of the pedigrees with three cases of CM, the mutation L94Q was detected, a mutation that has not previously been described in other populations [12].

Two families in our study were G101W positive. G101W is the most common missense mutation identified in melanoma pedigrees. This type of mutation is known as a founder mutation in our neighboring country Italy. The presence of the mutation in Slovenia could be the consequence of genetic material spreading through the geographical region [19]. In fact, one family did have an Italian ancestor.

All the mutations discovered in the patient group were located on exons 1α and 2, there were no mutations found on exon 1β. Mutations located on exon 1β affecting only p14ARF are generally rare [20,21]. In our study, the only allele variant detected on the exon 1β – F23F was detected in one of the individuals from the control group.

Family history and the presence of mutation are usually related to an earlier onset of the disease. The p16INK4A mutation-carriers in our study developed CM earlier than the patients without mutation, but the difference did not reach statistical significance (p = 0.257). This could be explained by the fact that all of the individuals with CM (both mutation positive and negative) had a family history of CM or multiple primary CM.

According to the literature, individuals carrying p16INK4A mutations are at a higher risk for developing a PC [6]. There were 4 CM/PC families in our study. In one of these families the variant A148T was detected. This variant was described in previously published studies as a polymorphism without any deleterious influence on the p16INK4A protein [22]. One study reported that A148T heterozygous carriers were more likely to have a first-degree relative with cancer of any type [23].

There were no deleterious *CDKN2A *mutations found in the three children, but all of them were positive for 500C>G and -191MetA>G variants. There were also no mutations found in the study published by Whiteman, so the conclusion is that other genes are important in the genesis of an early-onset melanoma [24].

We included in the study 26 patients with multiple primary CM without family history of the disease. Multiple primary CM could alone indicate an underlying genetic predisposition [15,25]. In the group of p16INK4A mutation carriers, six (6/13) patients indeed developed multiple primary CM. Only one patient without family history tested positive for possible p16INK4A mutation, in this case D84N. Many patients with multiple primary tumors were carriers of at least one of the 5'-UTR or 3'-UTR variants (18/26). There were also two carriers of the exon 2 allelic variant (A148T) in the group. The remaining eight patients with multiple primary CM had no changes in the *CDKN2A *sequence (8/26).

To the best of our knowledge there are no previous descriptions of the D84N variant. It is part of the coding region of the *CDKN2A *gene and as such may affect the protein function.

There are numerous reports about the prevalence of 3'UTR variants in the population of patients with CM, but the conclusions drawn in these studies are sometimes contradictory [23,26,27].

The prevalence of -191MetA>G did not differ between the group of CM patients and the control group in our study, which could confirm previous reports about low significance of that allelic variant in development of CM [28]. Although some authors report a higher prevalence of 500C>G in the population of CM patients and possible influence on the course of the disease, we also found high prevalence in the group of healthy individuals (19/64 vs. 13/54) [27]. The only observed difference between the group of healthy individuals and the patients with CM was the presence of the T allele (or 540C>T heterozygous carriers, 11/64 vs. 2/54). The heterozygous carriers were more numerous in the group of patients but after applying Bonferroni correction to account for multiple tested groups, the difference was statistically insignificant. A similar observation was reported by Kumar in the group of patients with sporadic CM [26]. In the study performed on Latvian sporadic CM patients, no changes in the frequency of both 3'UTR allelic variants were observed. The investigated polymorphic variants could have some influence on the CM risk by altering the level of *CDKN2A *transcription or by being in linkage disequilibrium with some unknown high-risk variant which could be the subject of further investigations [29].

We did not discover a pedigree with *CDK4 *mutation in our population of familial CM, but this was expected since these mutations are very rare and affected families usually have numerous cases of CM [5,30].

The search for the *MC1R *variants led to results that were in conjunction with recently published reports about allele frequency across different populations [31]. Interestingly, the most common variant in our population was T314T with as high a frequency as in French and Dutch populations. Among all of the studied populations, V92M was one of the most common variants and this was also the case in our results, but the frequency was somewhat higher than that reported for Italian or Greek populations. V60L proved to have the same frequency as V92M and similar frequencies as in Dutch and French populations but higher than in Italian and Greek studies. We discovered rather high frequencies of RHC variants R151C and R160W, but only one case of D294H. The evaluation of allele frequencies by Gerstenblith presented significantly different distribution of *MC1R *variants across Caucasians. The allele frequency of combined RHC variants showed a geographic gradient with decreasing frequency from North to South with frequencies of 21.5%, 8.5% and 2.9% in Britain/Ireland, Italy and Greece, respectively [31]. The results from our population rank us somewhere in between and they probably reflect our geographical position and intensive migrations in the past. Frequencies of the most frequent allelic variants were slightly higher in the group of patients and their relatives than in the group of controls but the differences did not reach statistical significance. Four individuals with p16INK4A mutation had also one of the "R" alleles. Two of them developed CM but two are currently healthy. Based on this, no conclusions can be made about the influence of "R" alleles on p16INK4A penetrance [9]. The effect of discovered *MC1R *variants on the individual's pigmentation characteristics, or risk of skin cancer, remains to be elucidated.

## Conclusion

The present study has shown a high prevalence of *CDKN2A *mutations affecting p16INK4A in Slovenian population of familial melanoma patients (37%) and an absence of p14ARF or *CDK4 *mutations. This indicates the need to continue with the study of melanoma susceptibility genes in the Slovenian population in order to design effective prevention programs.

## Competing interests

The authors declare that they have no competing interests.

## Authors' contributions

BP coordinated the accrual of the participants, contributed to molecular genetic studies, performed the statistical analysis and data interpretation and drafted the manuscript. PC performed sequencing and sequence analysis, data interpretation and helped to draft the manuscript. SN participated in the study design and molecular genetic studies and has been involved in the approval of the final version. JZ participated in study design and critically revised the manuscript. NB participated in study design and gave critical remarks. MH conceived the study, was involved in drafting and critical revision of the manuscript, and gave final approval of version to be published.

## Pre-publication history

The pre-publication history for this paper can be accessed here:


